# Effect of Interfacial Polarization and Water Absorption on the Dielectric Properties of Epoxy-Nanocomposites

**DOI:** 10.3390/polym9060195

**Published:** 2017-05-28

**Authors:** Philipp Marx, Andrea J. Wanner, Zucong Zhang, Huifei Jin, Ioannis-Alexandros Tsekmes, Johan J. Smit, Wolfgang Kern, Frank Wiesbrock

**Affiliations:** 1 Polymer Competence Center Leoben (PCCL), Roseggerstraße 12, Leoben 8700, Austria; philipp.marx@pccl.at (P.M.); andreajohanna.wanner@pccl.at (A.J.W.); zucong.zhang@pccl.at (Z.Z.); wolfgang.kern@unileoben.ac.at (W.K.); 2 Chair of Chemistry of Polymeric Materials, Montanuniversitaet Leoben, Otto-Glöckel Straße 2, Leoben 8700, Austria; 3 Department of Electrical Sustainable Energy, Delft University of Technology, Mekelweg 4, Delft CD2628, The Netherlands; h.jin@tudelft.nl (H.J.); i.a.tsekmes@tudelft.nl (I.-A.T.); j.j.smit@tudelft.nl (J.J.S.); 4 Prysmian Cables and Systems B.V., Schieweg 9, Delft AN2627, The Netherlands

**Keywords:** epoxy resins, nanoparticles, surface functionalization, silylating agent, water uptake, permittivity, loss factor, interfacial polarization, thermal conductivity

## Abstract

Five types of nanofillers, namely, silica, surface-silylated silica, alumina, surface-silylated alumina, and boron nitride, were tested in this study. Nanocomposites composed of an epoxy/amine resin and one of the five types of nanoparticles were tested as dielectrics with a focus on (i) the surface functionalization of the nanoparticles and (ii) the water absorption by the materials. The dispersability of the nanoparticles in the resin correlated with the composition (OH content) of their surfaces. The interfacial polarization of the thoroughly dried samples was found to increase at lowered frequencies and increased temperatures. The β relaxation, unlike the interfacial polarization, was not significantly increased at elevated temperatures (below the glass-transition temperature). Upon the absorption of water under ambient conditions, the interfacial polarization increased significantly, and the insulating properties decreased or even deteriorated. This effect was most pronounced in the nanocomposite containing silica, and occurred as well in the nanocomposites containing silylated silica or non-functionalized alumina. The alternating current (AC) breakdown strength of all specimens was in the range of 30 to 35 kV·mm^−1^. In direct current (DC) breakdown tests, the epoxy resin exhibited the lowest strength of 110 kV·mm^−1^; the nanocomposite containing surface-silylated alumina had a strength of 170 kV·mm^−1^. In summary, water absorption had the most relevant impact on the dielectric properties of nanocomposites containing nanoparticles, the surfaces of which interacted with the water molecules. Nanocomposites containing silylated alumina particles or boron nitride showed the best dielectric properties in this study.

## 1. Introduction

Epoxide resins are among the most commonly used polymer-based insulating adhesives in high-voltage insulations [1,2]. These epoxy resins can be cured by anhydrides or amines after thermal activation. Concomitant with a more compact design of high-voltage machinery such as transformers and generators and originating from the higher power-to-volume ratio, all materials used for the assembly of high-voltage machinery must meet higher standards; in particular, higher thermal conductivity and increased AC/DC breakdown strength. Epoxy-based composites with inorganic fillers have been found to meet these demands [3,4,5,6,7], and, hence, in the last two decades, the design of composite materials comprised of either micro-scaled or nano-scaled inorganic particles has gained increased attention. Inorganic fillers such as AlN, BN, SiO_2_, Al_2_O_3_, TiO_2_, SiC, ZnO, etc. [8,9,10] have been used for the fabrication of electrically insulating polymers, aiming to deliver the targeted electrical, mechanical, and thermal properties.

As the thermal conductivity of amorphous organic polymers and resins in the range of 0.2 W·m^−1^·K^−1^ is very low, organic-inorganic composites/mixtures containing inorganic fillers with high thermal conductivity are frequently used to improve heat dissipation. As insulating properties are required, metal powders such as Cu or carbon allotropes such as graphite and graphene may not be considered as thermally conductive fillers. Comparably high values for thermal conductivity have been reported for (bulk) hexagonal BN (boron nitride) with λ = 390 W·m^−1^·K^−1^ [11]. If, in addition, such nanocomposites show high resistance to partial discharges, they may be classified as high thermal conductivity insulation systems with enhanced electric breakdown strength.

Previous studies and results suggested that the unique properties of polymers and the corresponding nanocomposites used as dielectrics originate from interfacial phenomena [12,13,14]. Several publications indicated that self-assembly is a crucial process in the formulation of nanocomposites; nanocomposites contain nanofillers, which should be homogeneously dispersed in the polymer matrix. Hence, in order to understand the properties that emerge from the composition and structure of the nanocomposite, it is indispensable to investigate the interaction between the nanofillers and the polymer matrix. Among the polarization and relaxation phenomena to significantly influence the performance of a dielectric, interfacial polarization as well as α and β relaxations are the most important [15]. Notably, while interfacial polarization originates from the structural inhomogenities of a material, this type of polarization is easily affected by (absorbed) water molecules [16]. In order to investigate these phenomena with a dedicated focus on the surface functionalization of the nanoparticles on the one hand and the effect of water absorption by the materials on the other, the choice of materials and the material properties listed hereafter were combined for the investigations summarized in this article.

**Scheme 1:**
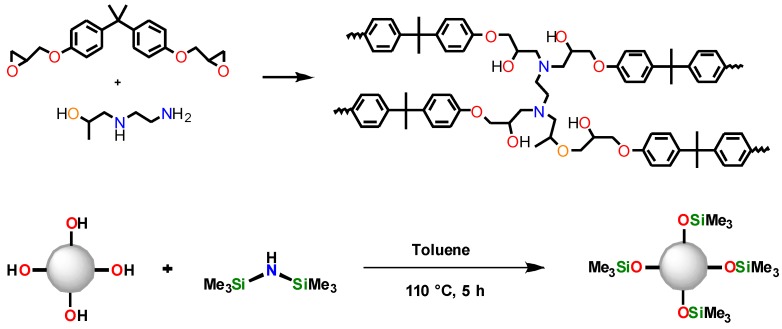
**Top**: Schematic representation of the ring-opening crosslinking reaction of Bisphenol A diglycidyl ether (DGEBA) with 1-(2-aminoethylamino)-2-propanol (AEAP); **Bottom**: Schematic representation of the surface functionalization of inorganic nanoparticles bearing M–OH groups with hexamethyl disilazane (HMDS).

Choice of materials: In this study, epoxy/amine resins composed of the bis-epoxy compound Bisphenol A diglycidyl ether (DGEBA) and the tetra-functional amine/alcohol hardener 1-(2-aminoethylamino)-2-propanol (AEAP) were chosen (Scheme 1). This resin has a glass-transition temperature of 78 °C in the cured state [17]. As nanofillers, silica, alumina, and hexagonal BN were chosen. As the silica and alumina nanoparticles contained M–OH groups on their surface (see Section 3.1), they were functionalized by the reaction with hexamethyl disilazane to yield trimethyl silyl (TMS) ethers (Scheme 1, bottom). Both types of silica and alumina particles, the non-functionalized as well as the functionalized ones, were used as nanofillers in this study, yielding a total of six types of specimens that were investigated; the unfilled epoxy resin (referred to as DGEBA) as well as the nanocomposites containing non-functionalized silica (SiO_2_), functionalized silica (SiO_2_-TMS), non-functionalized alumina (Al_2_O_3_), functionalized alumina (Al_2_O_3_-TMS), and (non-functionalized) boron nitride (BN).

Material properties: In order to correlate the dielectric properties of the nanocomposites with their composition, the following parameters were investigated: (i) chemical composition of the nanoparticles’ surfaces before and after functionalization; (ii) influence of nanofillers on the curing reaction; (iii) dispersability of the nanofillers in the polymer matrix; (iv) permittivity and dissipation factor of thoroughly dried samples; (v) permittivity and dissipation factor of samples stored under ambient conditions; (vi) AC/DC breakdown strength; (vii) quantification of the water uptake; and (viii) thermal conductivity.

## 2. Materials and Methods

### 2.1. Materials

Bisphenol A diglycidyl ether (DGEBA), 1-(2-aminoethylamino)-2-propanol (AEAP), and Al_2_O_3_ nanoparticles (particle sizes: 20–30 nm; surface area: 180 m^2^·g^−1^) were purchased from ABCR (Karlsruhe, Germany). SiO_2_ nanoparticles (particle sizes: 5–15 nm; surface area: 590–690 m^2^·g^−1^) and hexamethyldisilazane (HMDS) were obtained from Sigma Aldrich (Vienna, Austria). Hexagonal BN nanoparticles MK-hBN-N70 (average size: 70 nm; surface area: 45 m^2^·g^−1^) were purchased from MK Impex Corporation (Missisauga, ON, Canada). The solvents toluene and dichloromethane were purchased from Carl Roth (Vienna, Austria) and VWR (Vienna, Austria), respectively. The release agent Chemlease R&B EZ (1116650226) was acquired from Chem Trend GmbH (Maisach-Gernlinden, Germany). All chemicals were used as received.

### 2.2. Instrumentation

FT-IR spectra were recorded on a Bruker Vertex 70 FT-IR spectrometer (Bruker Austria GmbH, Vienna, Austria), using an ATR unit over a spectral range from 600 to 4000 cm^−1^ with a resolution of 4 cm^−1^. For each sample, a background correction was performed and 16 scans were recorded. TGA single measurements were performed on a Mettler Toledo TGA/DSC 1 Star System (Mettler-Toledo GmbH, Vienna, Austria). The samples were heated from 25 to 900 °C with a heating rate of 12 K·min^−1^ and a nitrogen flow rate of 30 mL·min^−1^. ^1^H-NMR spectra were recorded on a Bruker 500 MHz nuclear magnetic resonance spectrometer (Bruker BioSpin Corporation, Billerica, MA, USA). For referencing, the residual solvent signal of CDCl_3_ at 7.26 ppm was used. Mass spectra were recorded using a Shimadzu Multi-Shot Pyrolyzer EGA/PY-3030D coupled to a Shimadzu GCMS-QP2010 Ultra GC-MS spectrometer (Shimadzu Deutschland GmbH, Duisburg, Germany). The sample was pyrolized by a single shot at 600 °C and subsequently analyzed over a range from 50 to 500 *m*/*z*. For the duplicate measurement of thermal conductivity, specimens with a diameter of 50 mm and a height between 2 and 3 mm were prepared. The samples were measured with a DTC-300 thermal conductivity meter (TA Instruments, New Castle, Delaware), according to the ASTM standard E1530 at temperatures of 30, 60, and 90 °C. DSC single measurements were performed on a Mettler Toledo DSC 822e Differential Scanning Calorimeter (Mettler-Toledo GmbH, Vienna, Austria). For the determination of the glass-transition temperature, the samples were heated twice from 0 to 275 °C with a heating rate of 20 K·min^−1^. For the measurement of the curing reaction, a freshly prepared stoichiometric mixture of DGEBA, a nanoparticle-DGEBA mixture, and AEAP was heated from 5 to 250 °C with a heating rate of 10 K·min^−1^. For the dielectric characterization, a Novocontrol Alpha-A dielectric analyser with a ZGS Alpha active cell (Novocontrol Technologies, Montabaur, Germany) and a temperature control unit (Quarto cryosystem) (Novocontrol Technologies, Montabaur, Germany) was used. For the measurements, specimens with a diameter of 40 mm and a height of 1 mm were prepared. The dielectric spectra were recorded twice; for thoroughly dried samples (5 days at 140 °C and reduced pressure) on the one hand and samples that were stored for 10 d under ambient conditions on the other. A frequency range from 10^−2^ to 10^6^ Hz at temperatures of −20, 0, 20, 40, and 60 °C was applied. The experiments were performed in quadruplicate. For the determination of the breakdown voltage, pre-dried specimens (three days at 100 °C) with diameters of 60 mm and heights of 1 mm were used. The specimens were placed between a spherical-shaped earth electrode (*r* = 26 mm) and a spherical-shaped high voltage electrode (*r* = 11 mm); during the measurements, the voltage was increased with a rate of 2 kV·s^−1^ (AC) and 5 kV·s^−1^ (DC), respectively. The measurements were performed with nine repetitions in mineral oil. For the water uptake study, specimens with a diameter of 60 mm and a height of 1 mm were used. After drying at 100 °C for 72 h, the samples were weighed and placed into a humidity chamber at 50 °C and 50% relative humidity. The water uptake was monitored gravimetrically; the experiments were performed in duplicate. TEM (transmission electron microscope) images were conducted on a FEI Tecnai 12 transmission electron microscope (FEI, Hillsboro, OR, USA). For sample preparation, ultra-thin sections were cut from the nanocomposites using a microtome equipped with a diamond knife at room temperature. For the dispersion of the nanoparticles in the resin, a Dissolver Dispermat AE 03 (20 min, 3000 rpm) (VMA-Getzmann GmbH, Reichshof, Germany) and a Bandelin Sonoplus HD 3200 ultrasonic device (amplitude 30%, pulse off) were used (Bandelin, Berlin, Germany).

### 2.3. Modification of the Nanoparticles

The surfaces of the SiO_2_ and Al_2_O_3_ nanoparticles were functionalized according to a general synthetic protocol; the nanoparticles (5.00 g) were dispersed in toluene (100 mL), and hexamethyldisilazane (HMDS) (20 mL) was added. The mixture was stirred at 110 °C for 5 h. After cooling down, the dispersion was centrifuged (4 min, 4000 rpm) and the obtained solid was washed with toluene five times. The product was dried at 110 °C for 48 h.

TMS-modified SiO_2_ nanoparticles: FT-IR (ATR): ν (cm^−1^) = 2962 (C–H), 1632 (H_2_O), 1255 (Si–CH_3_), 1062 (Si–O–Si), 948, 847, 802, 758. ^1^H-NMR (500 MHz, CDCl_3_): δ (ppm) = 0.16 (s, 9H, Si(CH_3_)_3_). MS (*m*/*z*): 75.05 [C_2_H_7_SiO^+^].

TMS-modified Al_2_O_3_ nanoparticles: FT-IR (ATR): ν (cm^−1^) = 2958 (C–H), 1633 (H_2_O), 1253 (Si–CH_3_). ^1^H-NMR (500 MHz, CDCl_3_): δ (ppm) = 0.16 (s, 9H, Si(CH_3_)_3_). MS (*m*/*z*): 75.05 [C_2_H_7_SiO^+^].

### 2.4. Compounding of the Nanocomposites

For the compounding of the nanocomposites, 10.21 g of the corresponding nanoparticles were added to 174 g of DGEBA. For a homogeneous distribution of the nanoparticles in the polymer matrix, the mixture was stirred with a high-shear mixer (20 min, 3000 rpm) at room temperature and sonicated (amplitude 30%, puls off) for 15 min while being cooled with an ice bath.

### 2.5. Preparation of the Test Specimens

For the preparation of the test specimens containing 5 wt % of nanoparticles, a nanoparticle-DGEBA dispersion (see Section 2.4.) was mixed with AEAP in a mass ratio of 6.21:1 (for the preparation of unfilled specimens, DGEBA was mixed with AEAP in a mass ratio of 5.76:1; the molar ratio of DGEBA:AEAP was 2:1 in all cases). In order to decrease the viscosity of the mixtures during the filling of the steel templates, 20 wt % of dichloromethane were added, and the mixture was stirred at room temperature for 5 min. The mixture was poured into a circular steel mould, which was coated with the release agent Chemlease R&B EZ. The solvent was removed under reduced pressure (0.1 mbar) at room temperature for 30 min. Afterwards, the resin was cured at 80 °C for 3 h. Specimens with the following geometries were prepared: diameter = 40 mm, height = 1 mm (for permittivity measurements); diameter = 60 mm, height = 1 mm (for electrical breakdown measurements); and diameter = 50 mm, height = 2–3 mm (for thermal conductivity measurements).

## 3. Results

### 3.1. Surface Functionalization of the Nanoparticles and Preparation of the Nanocomposites

**Figure 1:**
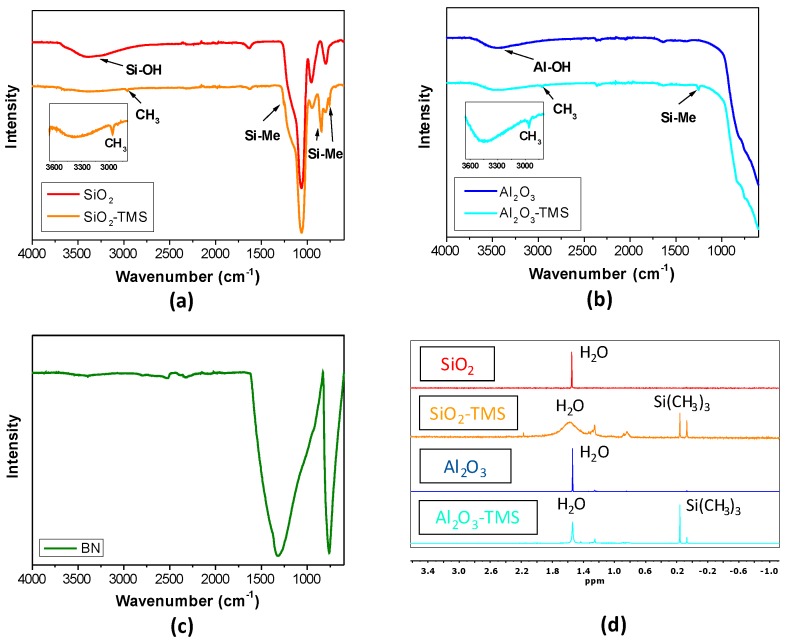
IR spectra of pristine and functionalized silica (**a**) as well as alumina (**b**) and non-functionalized BN (**c**) as well as the ^1^H-NMR spectra of the pristine and functionalized silica and alumina particles (**d**); ”H_2_O” acronyms all inorganic “OH” groups.

The epoxy/amine resin composed of DGEBA and AEAP was used as the polymer matrix in this study, in which the effect of nanoparticles on the water uptake and the dielectric properties of the nanocomposites were investigated. AEAP contains one primary and one secondary amine, as well as one hydroxy group (the latter with an autocatalytic effect on the curing reaction [17]; Scheme 1, top). Notably, the primary amine reacts faster than the secondary amine; the secondary OH groups (yielded by the ring-opening of the epoxides; Scheme 1) only react at high temperatures, while, on the other hand, the primary alcohol function of the AEAP can competitively react with the epoxides [18]. Hence, it was considered that AEAP has four (potential) reaction centers for the crosslinking reaction with bisfunctional DGEBA. Three types of nanoparticles were chosen for this study; namely, silica, alumina, and (chemically “inert”) hexagonal BN. Unlike BN, alumina and silica particles bear a varying content of hydroxyl groups on their surface. This study aimed, among other things, at investigating the interaction of nanoparticles with the polymer matrix in which they are embedded. Hence, the nanoparticles used for the preparation of the nanocomposites were used as received on the one hand and, on the other, surface-treated with the silylating compound HMDS, aiming at the provision of surfaces with reproducible composition and significantly lowered amounts of hydroxyl groups present. As a silylating agent, HMDS was chosen, which reacts with water adsorbed on the particles’ surfaces and subsequently converts the hydroxy groups into trimethyl siloxy functions (Scheme 1) [19]. In this highly reproducible process, HMDS alters the hydrophilic properties of the prisitne nanoparticles into hydrophobic ones, yielding trimethyl silyl-functionalized alumina (Al_2_O_3_-TMS) and silica particles (SiO_2_-TMS). The successful functionalization of the surfaces of the silica and alumina nanoparticles was monitored by IR (revealing C–H and Si–CH_3_ absorption bands of the functionalized silica and alumina particles, as well as the reduced amount of OH groups; there was not any significant amount of OH groups in BN nanoparticles; Section 2.3 and Figure 1) and ^1^H-NMR analyses of dispersions of the nanoparticles (presence of the Si–(CH_3_)_3_ signal; Section 2.3; Figure 1), as well as mass spectrometry (detection of the C_2_H_7_SiO^+^ fragment; Section 2.3).

**Figure 2:**
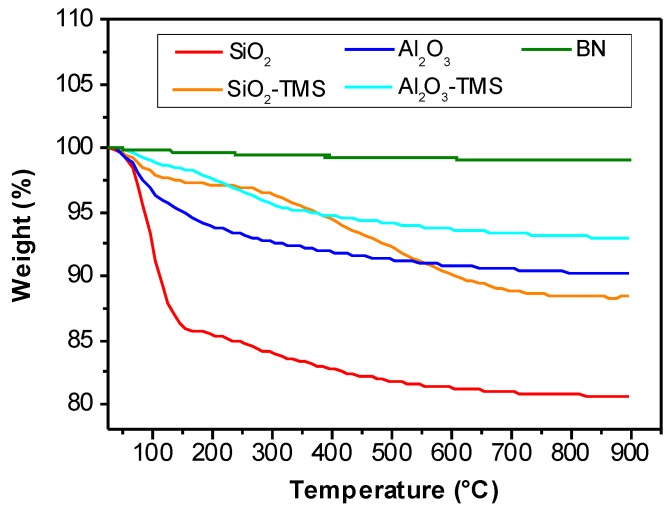
Thermograms of the nanoparticles used in this study.

In addition, thermogravimetric analyses were performed with unreacted as well as trimethyl silyl-functionalized alumina and silica nanoparticles (Figure 2; BN did not show any weight loss in the temperature range). The data show a pronounced weight loss in the range of 15 wt % of the pristine silica at temperatures below 200 °C, which originates from the evaporation of surface-adsorbed water molecules. Upon continued heating to temperatures higher than 200 °C, the weight loss increases to a total of 20 wt %, which is indicative of the condensation of adjacent silanol groups yielding silyl ethers. The weight loss of unreacted alumina starts in the same temperature range as silica but amounts to a total of approx. 5 wt % only, which is indicative of lower amounts of aluminum hydroxide groups present at the surface (compared to silica). Unlike silica, the thermograms of alumina do not reveal a second step of weight loss starting at temperatures higher than 200 °C, which means that the condensation of aluminum hydroxide groups does not play a significant role in the temperature range until 900 °C. Hence, it may be argued that the silica particles have a higher content of hydroxy groups on and larger amounts of water adhered to their surfaces than the alumina particles do. The trimethyl silyl-functionalized particles have significantly lower amounts of water adsorbed, and, consequently, only show weight losses in the range of 1 (alumina) and 2.5 wt % (silica) for temperatures up to 200 °C. At elevated temperatures, the trimethyl silyl groups are cleaved from the particles and, overall, they show a weight loss of 10 wt % in the case of silica and 5 wt % in the case of alumina (starting from approx. 250 °C). The higher total weight loss in the case of functionalized silica particles (compared to functionalized alumina particles) can be explained by a higher degree of functionalization (a larger number of trimethyl silyl groups per gram) due to the larger number of hydroxyl groups (see above).

**Figure 3:**
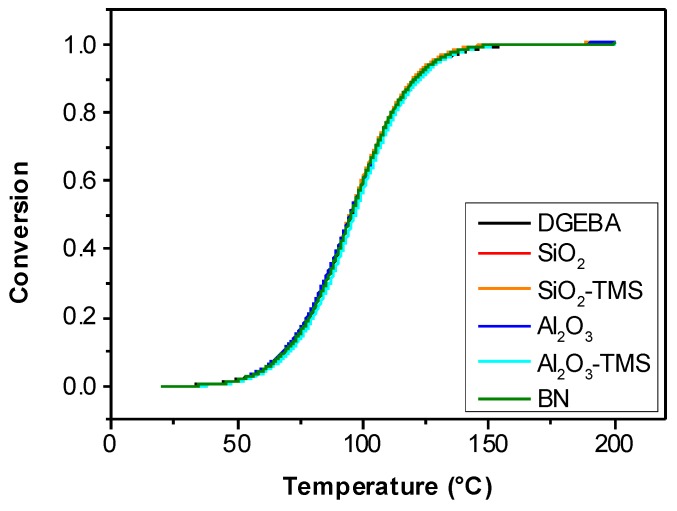
DSC for the monitoring of the curing of the epoxy resin, Bisphenol A diglycidyl ether (DGEBA), as well as the nanocomposites.

For the production of nanocomposites, mixtures of DGEBA and AEAP in a stoichiometric 2:1 ratio (mol:mol) and 5 wt % of the respective nanoparticles were thoroughly mixed using mechanical stirring and ultrasonic treatment of the mixture prior to pouring them into steel molds of the targeted geometry and thermal curing at 80 °C for 3 h. The curing kinetics were monitored by differential scanning calorimetry with a heating rate of 10 K·min^−1^, starting at room temperature (Figure 3). The curing rate was found not be altered by the presence or absence of nanoparticles. Notably, even non-surface functionalized silica did not accelerate the curing reaction, despite the huge abundance of hydroxy groups with a potential autocatalytic effect (either as functional groups on the nanoparticles’ surfaces or in the adsorbed water molecules). The epoxy resin as well as all five types of nanocomposites showed the same curing rate and reached the same extent of curing.

**Figure 4:**
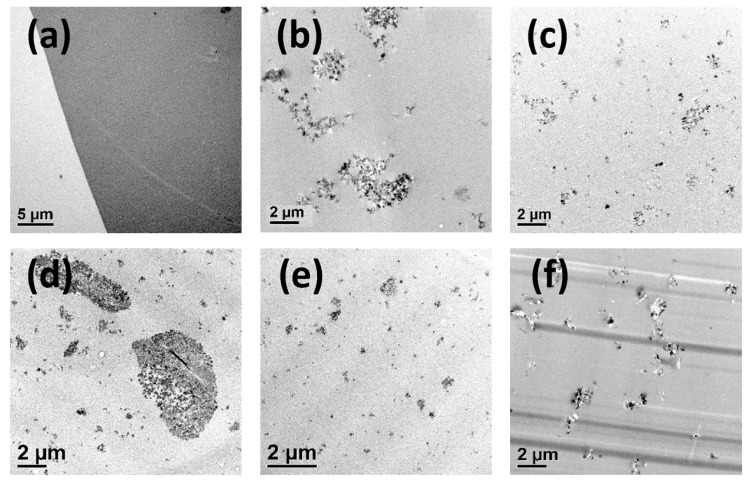
Transmission electron microscopy images of the (**a**) unfilled epoxy resin and the nanocomposites containing; (**b**) SiO_2_; (**c**) SiO_2_-TMS; (**d**) Al_2_O_3_; (**e**) Al_2_O_3_-TMS; and (**f**) BN.

The dispersion of the nanoparticles in the epoxy resin matrix was investigated by TEM images of cross sections of the specimens (Figure 4). Large aggregates of nanoparticles were found in the nanocomposites containing non-functionalized alumina and silica particles (Figure 4b,d). On the contrary, the surface-functionalized analogues (Figure 4c,e) were more uniformly dispersed in the polymer matrix, resulting in the formation of smaller aggregates. Hexagonal BN particles (Figure 4f), which exhibit only low amounts of hydroxy groups on their surface, showed similar dispersion to the surface-functionalized silica and alumina particles.

### 3.2. Permittivity Measurements of the Unfilled Epoxy Resin and the Nanocomposites

The relative permittivity is one key parameter to quantify the electric properties of a material. It is calculated as the ratio of the capacitance of a capacitor filled with a dielectric material divided by that of the same capacitor in vacuum. In the cases of polymer materials and their corresponding nanocomposites, it must be carefully considered that the relative permittivity increases with the polarizability of the material and its ability to store charges. While the polarization of a given material does not react to external stimuli instantaneously, a phase shift occurs with respect to the response; correspondingly, the relative permittivity is best described as a complex function (Equation (1); ε’_R_: real part of the permittivity; ε’’_R_: imaginary part of the permittivity). Both the real and the imaginary part of the complex permittivity vary with the temperature and the frequency.

ε_R_ = ε’_R_ − i·ε’’_R_ (1)

**Figure 5:**
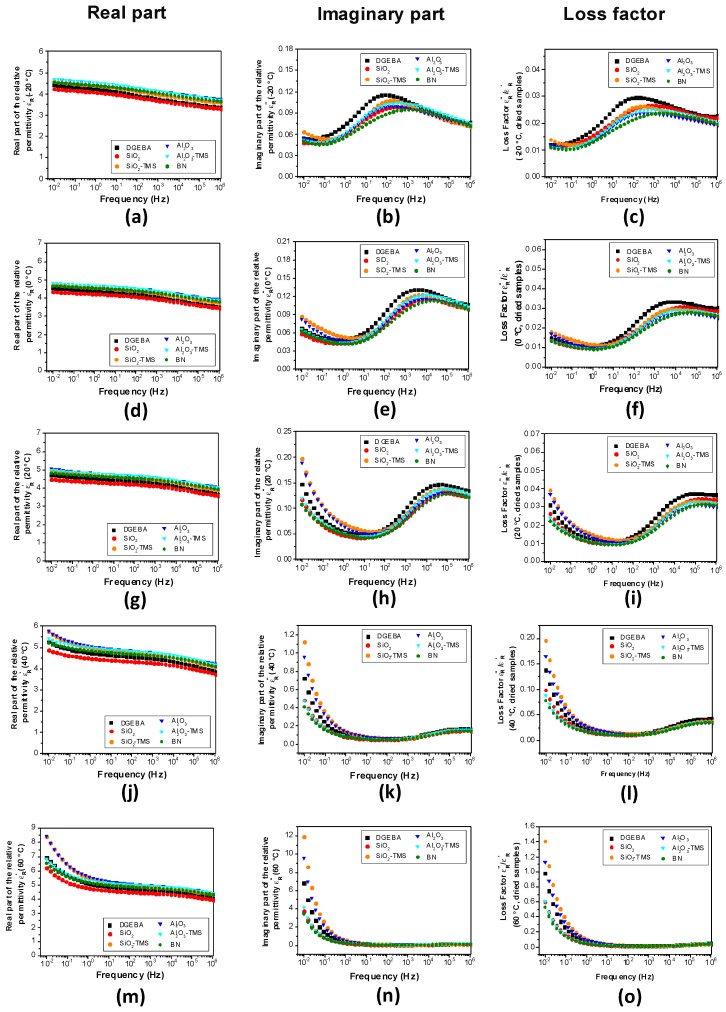
Real part (**a**,**d**,**g**,**j**,**m**) and imaginary part (**b**,**e**,**h**,**k**,**n**) of the permittivity as well as the loss factor (**c**,**f**,**i**,**l**,**o**) of the epoxy resin and the nanocomposites at −20 °C (**a**,**b**,**c**), 0 °C (**d**,**e**,**f**), 20 °C (**g**,**h**,**i**), 40 °C (**j**,**k**,**l**), and 60 °C (**m**,**n**,**o**).

**Figure 6:**
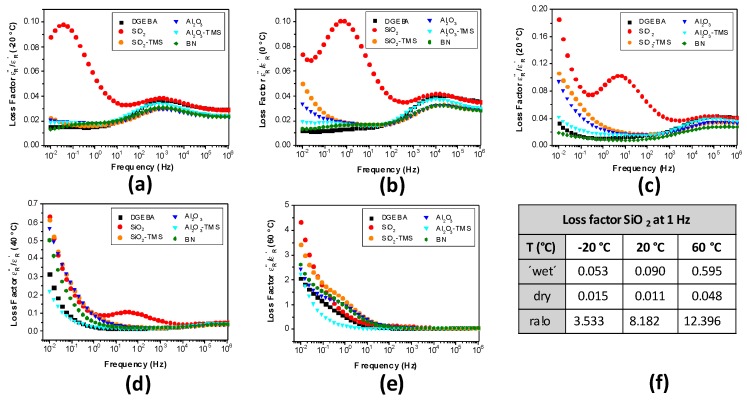
Loss factor of the epoxy resin and the nanocomposites at −20 °C (**a**), 0 °C (**b**), 20 °C (**c**), 40 °C (**d**), and 60 °C (**e**). A comparison of the loss factor of non-functionalized silica in dry and ”wet” states is provided in the inserted table (**f**).

The relative permittivity of the epoxy resin and the five different nanocomposites has been measured at five different temperatures, namely −20, 0, 20, 40, and 60 °C, and frequencies ranging from 10^−2^ to 10^6^ Hz (Figure 5). The permittivity at 50 Hz (Table 1) of all specimens increases with the temperature. At 20 °C, the permittivity spans a range of 4.2–4.7, which is in the upper range of values to be expected for epoxy resins [14]. The real part of the permittivity of all nanocomposites as well as the unfilled epoxy resin shows very comparable trends over the range of measurements (Figure 5). At −20, 0, and 20 °C, the frequency hardly influences the real part of the permittivity, which ranges from 4 to 5. At 40 and 60 °C, the real part of the frequency increases for frequencies lower than 10 Hz. Large amounts of water can be excluded to cause the polarization phenomena as the specimens were thoroughly dried prior to the measurements (see also Figure 6). As the real part of the permittivity increases only at elevated temperatures, vibrations of the chemical network constituting the polymer matrix are likely to cause these phenomena.

At each temperature, the following order of the permittivity (with respect to the nanoparticles) can be observed: SiO_2_ < (unfilled resin) < SiO_2_-TMS ≈ BN < Al_2_O_3_ ≈ Al_2_O_3_-TMS. A possible explanation for this unexpected behavior (which, by the way, is true for all temperatures and frequencies employed in this study) can be derived from the Tanaka model of nanocomposites, which distinguishes between three interaction zones of nanoparticles and polymer matrices in composite materials; a first and second layer (both of them “bound”) as well as a third “loose” layer [20]. The direct interaction of the functional groups of the polymer matrix (Scheme 1) and the surface of the nanoparticles is enabled in the first layer. Three types of surfaces are provided by the nanoparticles, namely, pronouncedly negatively charged surfaces of pristine silica [21], “chemically inert or quasi-inert” surfaces of highly silylated silica [22] and BN, as well as amphoteric surfaces of alumina [23]; as alumina can be functionalized only to low extent (Figure 2), no distinction between pristine and functionalized alumina needs to be made. A highly abundant functional group of the polymer matrix is the alcohol group (Scheme 1), the proton of which may interact in proton bridges with highly electronegative atoms, most easily accessible on the negatively charged silica surface. On the contrary, at pH = 7, the surface of alumina is charged positively and, hence, repels acidic protons. These three different types of interactions occur in the first layer (Tanaka model) and affect the second and third layer, which contribute to the increase or reduction of the permittivity. Intense interaction of the silica particles and the polymer matrix disturbs the mobility of dipoles in the second layer, which decreases the permittivity.

Unlike the real part of the permittivity, the imaginary part of the permittivity shows a pronounced dependence on the frequency applied (Figure 5). These effects are reproduced in the frequency dependency of the loss factor (Figure 5), which is calculated as the ratio of the imaginary and the real part of the permittivity (Equation 2). While ε’_R_ indicates the storage capacity of the material, the imaginary part ε’’_R_ quantifies the losses of energy. Hence, the conductivity of a material can be described by the loss factor tan δ. An ideal insulator would show no loss of energy (tan δ = 0), and materials with tan δ << 1 can be considered good dielectrics.

tan δ = ε’’_R_/ε’_R_ (2)

In the lower temperature range, precisely during the measurements at −20, 0, and 20 °C, the loss factor exhibits low values (tan δ < 0.04) over the whole range of frequencies, which is indicative of good insulating properties. At this range of temperatures, the loss factor has a local maximum in the frequency range from 1 to 10^6^ Hz, which shifts from approx. 0.1 kHz (−20 °C) via 3 kHz (0 °C) to 70 kHz (20 °C). This correlation of the loss factor with the frequencies applied may be referred to the β relaxation of rigid structural segments of the polymer network containing OH groups (Scheme 1). Notably, this effect of β relaxation is shifted to higher frequencies and is observable to a lesser extent (lower value of tan δ) upon the addition of nanofillers (unfilled polymer matrix versus nanocomposites), which can be explained by the interactions of the nanoparticles and the polymer matrix (see above: Tanaka model). Starting from 1 Hz, the loss factors increase in the case of decreasing frequency, which is indicative of interfacial polarization likely to originate from structural inhomogenities. At elevated temperatures of 40 and 60 °C, the loss factor is comparably high, in particular for frequencies lower than 10 Hz, at which interfacial polarization occurs. The effect of β relaxation plays a minor role at these elevated temperatures compared to interfacial polarization. Notably, all measurements were performed at temperatures lower than the glass-transition point. The loss factor of all specimens at 50 Hz (industrial standard; Table 2), however, is significantly lower than 1, revealing that the epoxy resin as well as the nanocomposites may be classified as well-suited dielectrics. Due to the pronounced effects of interfacial polarization on the permittivity, the effect of water absorbed during storage of the samples under ambient conditions for 10 d was investigated as well. (The water uptake of the epoxy resin and the nanoparticles in a climate chamber was quantified in an additional experiment; Section 3.4.) Special attention was paid to the effect of water absorption on the loss factor (Figure 6).

As a general trend, the loss factors of the “wet” specimens (Figure 6) that were stored under ambient conditions were found to be higher than those of their thoroughly dried counterparts. This trend becomes most easily discernible for all types of specimens for frequencies lower than 10^2^ Hz. Nanocomposites containing non-functionalized silica particles show the most dramatic change of the loss factor in that range of frequencies; the loss factor of the samples stored under ambient conditions is up to 12-times higher than that of thoroughly dried samples. The loss factor of all other specimens shows a less pronounced effect upon storage under ambient conditions; only in the case of the nanocomposites containing functionalized silica or non-functionalized alumina minor increases of the loss factor can be observed. These trends reflect the observations made during the thermogravimetric analyses (Figure 2), which revealed the rank order (with respect to the amount of adsorbed water) of non-functionalized silica >> non-functionalized alumina > functionalized silica > functionalized alumina. As the increases of the loss factor are most pronounced for frequencies lower than 10^2^ Hz, interfacial polarization is the likely phenomenon causing these trends, and it may be argued that the interactions of the nanoparticles’ surfaces with water are most intense in the case of silica (compared to alumina and BN).

### 3.3. Breakdown Voltage

**Figure 7:**
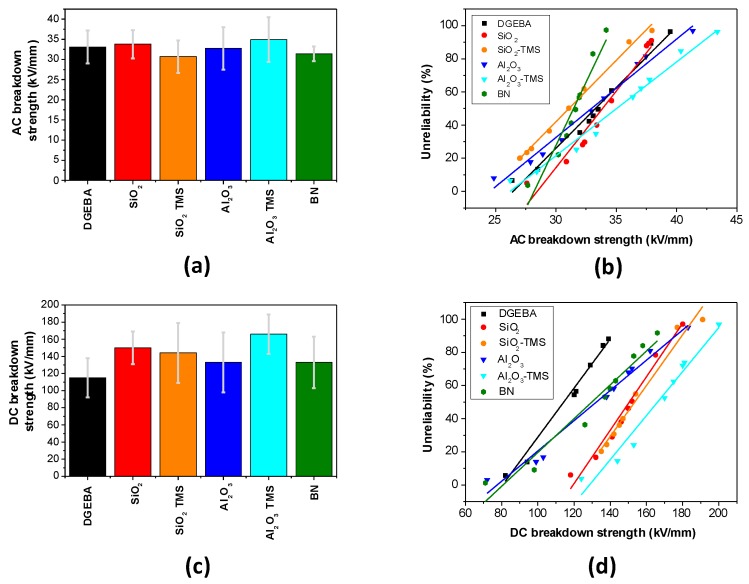
AC and DC breakdown strength of the epoxy resin and the nanocomposites (**a**,**c**) as well as the Weibull plots (**b**,**d**).

Thoroughly dried specimens were also tested in AC and DC breakdown tests in mineral oil (Figure 7). Their breakdown behaviour was analysed by Weibull plots (Figure 7; Table 3). Under alternating current, the nanocomposites did not behave differently within one standard deviation to the epoxy resin; all specimens showed a breakdown strength in the range of 30–35 kV·mm^−1^. On the contrary, under direct current, the epoxy resin exhibited the lowest breakdown strength of 110 kV·mm^−1^. Both types of silica, non-functionalized alumina, and BN showed slightly higher breakdown strength, though within one standard deviation. The nanocomposite containing trimethyl silyl-functionalized alumina has a breakdown strength of 170 kV·mm^−1^, which is out of the range of one standard deviation referred to the unfilled epoxy resin. A possible explanation for this targeted increase of the breakdown strength is the good dispersability of this type of nanoparticle in the epoxy matrix (Figure 4) in combination with the low tendency to adsorb water on the trimethyl silyl-functionalized surfaces and/or to interact with OH groups from water molecules and the resin, respectively.

### 3.4. Water Uptake

**Figure 8:**
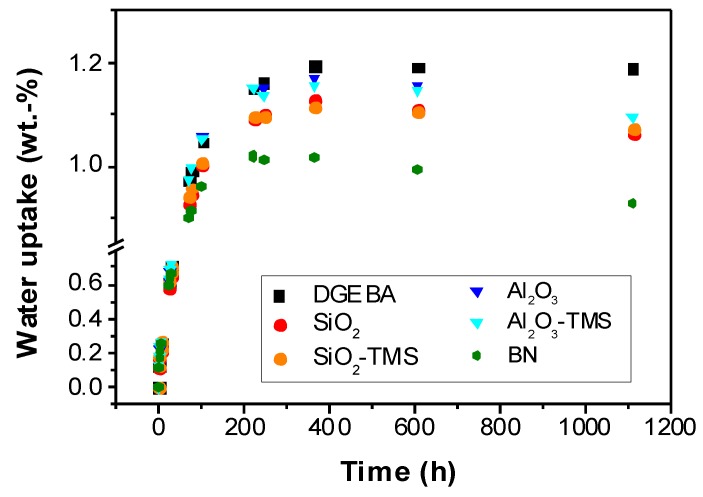
Water uptake study (50 °C, 50% relative humidity) of the epoxy resin and the nanocomposites over 1.5 months.

As the water uptake upon storage under ambient conditions was found to alter the permittivity of the specimens, the water absorption was quantified in a climate chamber operated at elevated temperatures of 50 °C and (standardized) 50% relative humidity for 6 weeks (Figure 8). Considering the final measurements as (quasi-) equilibrium conditions, three classes of water absorption can be found: the unfilled epoxy resin showed the highest absorption of 1.2 wt %, the nanocomposites containing any type of silica or alumina showed medium absorption of 1.1 wt %, and the nanocomposite containing BN showed the lowest absorption of 0.9 wt %. The nanoparticles were added in 5 wt %; considering the density of alumina (3.95 g·cm^−3^), silica (2.60 g·cm^−3^), BN (2.10 g·cm^−3^) [24] and the polymer (1.15 g·cm^−3^) [14], volume fractions of the nanoparticles of 2.0 (silica), 1.5 (alumina), and 2.7 vol % (BN) may be calculated. Hence, the similar water uptake of nanocomposites containing silica or alumina on the one hand and the low water absorption of the network containing BN on the other cannot be exclusively referred to the lower water adsorption by the nanoparticles (compared to the unfilled epoxy resin). Instead, also different degrees of water adsorption by the nanoparticles themselves must be considered: Alumina and silica interact more intensely with water, while BN does not adsorb any water (cp., Section 3.1 and Figure 2). While the crosslinked resin (with a surface energy of 37 mN·m^−1^) itself only absorbs water in the range of 1 wt %, the different degrees of interaction of non-functionalized and functionalized particles are not resolved in this water uptake study (all types of silica and alumina nanoparticles can adsorb more than 1 wt % of water). However, while the water uptake of the unfilled resin increased in the course of the 1200 h study until a maximum (equilibrium) of 1.2 wt % had been reached, the water uptake of all nanocomposites decreased after reaching a maximum value by up to 0.1 wt %. This observation can be explained by structural re-orientation of the nanoparticles in the polymer matrix that, due to nanoparticle-polymer interactions (Tanaka model) renders the network stiffer and, consequently, enables the uptake of reduced amounts of water only [25]. The permittivity of the “wet” nanocomposites (Section 3.2.) at 40 and 60 °C, respectively, reflects the effect of the assumed structural reorientation at the conditions of this study (50% r.h. at 50 °C).

### 3.5. Thermal Conductivity

**Figure 9:**
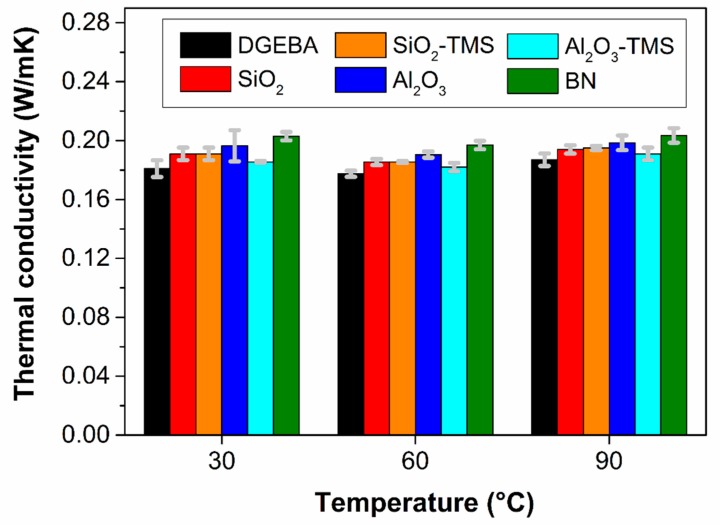
Thermal conductivity of the epoxy resin and the nanocomposite at 30, 60, and 90 °C.

At room temperature, the nanoparticles used in this study exhibit thermal conductivities of 0.7 (fused silica) [24], 23 (sintered alumina) [24], and 390 W·m^−1^·K^−1^ (bulk hexagonal BN) [11]. The thermal conductivity was measured on disc-shaped specimens with a height of 2–3 mm at temperatures of 30, 60, and 90 °C. The thermal conductivity of a given type of specimen was not significantly altered by the temperature, despite the literature reporting an increase of the thermal conductivity at elevated temperatures [26,27,28]. In each of the three sets of measurements (Figure 9), the unfilled epoxy resin always had the lowest thermal conductivity of approximately 0.18 W·m^−1^ K^−1^, while the nanocomposite containing hexagonal BN always had the highest thermal conductivity of approx. 0.20 W·m^−1^ K^−1^. Considering the standard deviations, only the nanocomposites containing hBN had a higher thermal conductivity than the unfilled resin and all other nanocomposites. Notably, in this study, this is the type of nanofiller with the largest particle size and the highest (bulk) thermal conductivity. By the addition of hBN nanofillers in 5 wt % quantities, the thermal conductivity could be increased by approximately 10%.

## 4. Discussion and Conclusions

In the course of this study, epoxy/amine resin-based nanocomposites were investigated as dielectrics and compared to the unfilled resin. Three types of commercially available nanoparticles were chosen (silica, alumina, and BN), two of which were reactive enough to enable surface functionalization by the reaction with hexamethyl disilazane. These five types of nanoparticles differed in the amount of hydroxy groups present at their surface (Figure 1) as well as the amount of water adsorbable on their surfaces (20% in the case of non-functionalized silica, 0% in the case of BN; Figure 2). In fact, the silica nanoparticles had a significantly higher amount of OH groups present on their surface than the alumina analogues; even the silylated silica nanoparticles still contained adsorbed water. While the nanoparticles did not alter the curing kinetics of the epoxy resin (Figure 3), the “OH-rich” (non-functionalized) silica and alumina nanoparticles exhibited aggregation in the polymer matrix, unlike the BN particles, the functionalized silica, and the alumina nanoparticles (Figure 4).

The detailed investigation of the electric and dielectric properties revealed that the interfacial polarization of the thoroughly dried samples increased at lowered frequencies and increased temperatures (Figure 5). The β relaxation of rigid structural segments of the polymer network, on the other hand, was not significantly increased at elevated temperatures. The extent of the interfacial polarization was lowered upon the addition of nanofillers (unfilled polymer matrix versus nanocomposites), which can be explained by the interactions of the nanoparticles and the polymer matrix. However, upon the absorption of water under ambient conditions, the interfacial polarization increased significantly and the insulating properties decreased or even deteriorated (Figure 6). This effect was most pronounced in the nanocomposite containing silica and occurred as well in the nanocomposites containing functionalized silica or non-functionalized alumina. It must be clearly stated that the nanocomposites containing “OH-rich” nanoparticles are not well-suited candidates for any application under ambient conditions, despite the fact that they can be thoroughly dried. Only the resin and the nanocomposites containing silylated alumina or BN perfectly act as dielectrics under ambient conditions. The water uptake study, on the other hand, revealed that the resin and the nanocomposites absorb water in a comparable range of approximately 1 wt % (Figure 8); hence, the deterioration of insulation properties originates not only from the amount of water absorbed but also from the interactions in the ternary resin-nanoparticle-water system. If water adheres to the nanoparticles’ surface, more intense polarization occurs, which decreases the insulating properties. Notably, the water uptake of the unfilled resin increased in the course of the 1200 h study until equilibrium had been reached; the water uptake of all nanocomposites, on the contrary, decreased after reaching a maximum value, which can be explained by the structural re-orientation of the nanoparticles in the polymer matrix due to nanoparticle-polymer interactions (Tanaka model).

The AC breakdown strength of all specimens was in the range of 30 to 35 kV·mm^−1^. In DC breakdown tests (Figure 7), the epoxy resin exhibited the lowest strength of 110 kV·mm^−1^; the nanocomposite containing surface-silylated alumina had strength of 170 kV·mm^−1^. The thermal conductivity (Figure 9) of the cured epoxy resin was 0.18 W·m^−1^·K^−1^ and was increased by max. 10% upon the addition of nanofillers.

In summary, for the application of nanocomposites as (high-voltage) insulators, the surface of the nanoparticles must be of a chemical composition such that they do not interact with water. The water absorption of the resin itself was found to have comparably little impact on the dielectric properties. Future studies will be performed at even higher temperatures, aiming at including the effect of α relaxation processes on the dielectric properties.

## Figures and Tables

**Table 1:** Permittivity (at 50 Hz) of the epoxy resin and the five different nanocomposites at −20, 0, 20, 40, and 60 °C.

Specimen ↓/T →	−20 °C	0 °C	20 °C	40 °C	60 °C
DGEBA	3.97	4.26	4.42	4.57	4.78
SiO_2_	3.86	4.01	4.22	4.35	4.52
SiO_2_-TMS	4.17	4.43	4.59	4.76	5.02
Al_2_O_3_	4.31	4.56	4.70	4.86	5.11
Al_2_O_3_-TMS	4.31	4.56	4.70	4.84	5.05
BN	4.20	4.42	4.55	4.69	4.89

**Table 2:** Loss factor (at 50 Hz) of the epoxy resin and the nanocomposites.

Specimen ↓/T →	−20 °C	0 °C	20 °C	40 °C	60 °C
DGEBA	0.029	0.020	0.013	0.012	0.018
SiO_2_	0.024	0.017	0.011	0.011	0.015
SiO_2_-TMS	0.025	0.016	0.012	0.014	0.024
Al_2_O_3_	0.023	0.015	0.011	0.013	0.023
Al_2_O_3_-TMS	0.023	0.015	0.010	0.010	0.016
BN	0.020	0.014	0.010	0.010	0.015

**Table 3:** Parameters of the Weibull analysis.

Specimen	AC Breakdown			DC Breakdown		
	β	η	*R*^2^	β	η	*R*^2^
**DGEBA**	9.635	34.828	0.978	6.806	124.306	0.988
**SiO_2_**	12.164	35.264	0.954	9.536	157.706	0.969
**SiO_2_-TMS**	8.098	32.470	0.994	7.970	162.367	0.971
**Al_2_O_3_**	7.311	34.884	0.982	4.869	146.065	0.977
**Al_2_O_3_-TMS**	7.723	37.192	0.981	9.432	175.415	0.961
**BN**	21.620	32.198	0.928	6.188	143.252	0.946
